# Understanding one health challenges in marginalized urban settings: A patient and public involvement (PPI) approach from the CHIP consortium activities across four global cities

**DOI:** 10.1016/j.onehlt.2024.100919

**Published:** 2024-10-18

**Authors:** Logan Manikam, Darlington David Faijue, Kalpita Shringarpure, Margi Sheth, Pam Factor-Livak, Priti Parikh, Hector Altamirano-Medina, Dewi Nur Aisyah, Radhika Sharma, Hemant Chaturvedi, Kaushik Sarkar, Rajib Dasgupta, Nancy Hiu Lan Leung, Pradeep Kumar Srivastava, Monica Lakhanpaul, on behalf of the Childhood Infection and Pollution (CHIP) Consortium

**Affiliations:** a Aceso Global Health Consultants Pte Limited, Singapore, Singapore; b Department of Epidemiology and Public Health, Institute of Epidemiology and Health Care, University College London (UCL), London, United Kingdom; c Institute for Infection and Immunity, St. George's, University of London, London, UK; d Department of Community Medicine, Medical College Baroda, Vadodara, Gujarat, India; e Department of Community Medicine, GCS Medical College, Hospital and Research, Ahmedabad, Gujarat, India; f Department of Epidemiology Columbia University Mailman School of Public Health, New York, USA; g University College London, Engineering for International Development Centre, Bartlett School of Sustainable Construction, Torrington Place, London, UK; h University College London, Bartlett School Env, Energy & Resources, London, UK; i Indonesia One Health University Network, West Jawa, Indonesia; j Jeevan Ashram Sanstha (JAS), Jaipur, India; k Aceso Global Health Consultants Limited, London, United Kingdom; l Institute for Health Modelling and Climate Solutions, Malaria No More, Washington DC, USA; m Population, Policy and Practice Department, UCL Great Ormond Street Institute of Child Health, 30 Guildford Street, London WC1N 1EH, UK; n Whittington Health NHS Trust, London, UK; o Centre of Social Medicine and Community Health, School of Social Sciences, Jawaharlal Nehru University, New Delhi, India; p WHO Collaborating Centre for Infectious Disease Epidemiology and Control, School of Public Health, Li Ka Shing Faculty of Medicine, The University of Hong Kong, Hong Kong Special Administrative Region; q National Vector Borne Disease Control Programme, M/o Health & FW, Govt of India, Res: Royal Villa-IV, 2/18 Flat 100, Sector-2, Rajendra Nagar, Sahibabad, Ghaziabad 201005, India

**Keywords:** One Health, Slum health, Patient and public involvement, Antimicrobial resistance, Qualitative research, Community health, Urbanization

## Abstract

**Background:**

Slum communities face health risks influenced by environmental, human, and animal health factors, particularly antimicrobial resistance (AMR). Tailored, community-driven solutions are needed to address these multifactorial health determinants. This study explores One Health challenges in urban slums using a Patient and Public Involvement (PPI) approach.

**Objectives:**

This study aims to use qualitative methods within a PPI framework to examine the social, environmental, and animal health factors contributing to AMR and other health challenges in urban slums. Focusing on One Health, we engaged slum residents in Jaipur, Jakarta, Antofagasta, and Istanbul through participatory approaches like social mapping and transect walks to identify health risks and develop intervention strategies.

**Methods:**

A PPI approach was employed to involve communities in the research process, ensuring culturally relevant insights. Data collection included social mapping, transect walks, and key informant interviews in the four cities, highlighting critical health determinants such as environmental contamination, healthcare access, and animal-related risks. Thematic analysis identified common challenges and intervention opportunities within the One Health framework.

**Conclusion:**

The study underscores the importance of PPI in addressing One Health challenges in urban slums and reveals interconnected human, environmental, and animal health risks. Engaging communities fostered trust and provided locally relevant solutions to complex health issues like AMR. Future interventions should be co-designed with communities to address social determinants like sanitation and healthcare access for sustainable outcomes.

## Highlights

• Community-driven One Health approach in urban slums.
• Research across Jaipur, Jakarta, Antofagasta, and Istanbul.
• Used social mapping, transect walks, and key informant interviews.
• Identified environmental, healthcare, and animal health risks.
• Emphasized co-designed, sustainable community health interventions.

## 1 Introduction

The rapid growth of urban populations has led to the proliferation of informal settlements [1], or slums, characterized by inadequate access to clean water, sanitation, and healthcare services [2]. These areas are hotspots for various health risks, particularly antimicrobial resistance (AMR) and infectious diseases, as residents face multiple socio-environmental challenges [2,3]. Urban slum environments present a unique confluence of challenges that demand an integrated approach to health research and intervention. Recent United Nations estimates suggest that the population of slum dwellers, defined as “highly populated, informal residential settlements characterized by insufficient services, shelter, sanitation, or amenities,” will reach two billion by 2030 [1]. Children in urban or peri-urban slums are particularly vulnerable to health risks [2]. This vulnerability is reflected in elevated infant mortality rates, which persist despite significant global efforts to reduce them [3]. Research by UNICEF indicates that urban children from the poorest segments in low-middle-income countries (LMICs) are more likely to die before the age of five and are less likely to complete primary school compared to their rural counterparts [4]. Poverty and poor living conditions are strongly associated with higher risks of diarrheal diseases [5] and debilitating infections among these children [6]. Additionally, they typically have limited access to healthcare and adequate nutrition [8], leading to long-lasting effects on their health and development and exacerbating community-wide inequalities and poverty cycles [9].

Addressing these health issues requires an integrated One Health approach that considers the interconnectedness of human, animal, and environmental health [10]. Engaging communities in the design and implementation of health interventions is essential for ensuring their relevance and success [7].

The One Health framework, an approach that recognizes the interconnectedness of human, animal, and environmental health [10], is particularly relevant in these settings. Slums are characterized by overcrowding, inadequate sanitation, and poor access to clean water [2], which can lead to increased disease transmission and resistance to antimicrobial treatments (AMR) [6]. In addition, slum dwellers often share spaces with animals and are exposed to high levels of pollution, creating a perfect storm for the spread of AMR and other health risks [6].

Previous studies have indicated that urban slums face disproportionate burdens of infectious diseases, environmental hazards, and limited access to healthcare [4]. Addressing these issues through a One Health lens involves looking beyond individual risk factors to consider the complex interactions between human, animal, and environmental health [10]. This paper presents a Patient and Public Involvement (PPI) approach to identifying One Health challenges in urban slums across four cities: Jaipur (India), Jakarta (Indonesia), Antofagasta (Chile), and Istanbul (Turkey). By involving slum residents in the research process, the goal was to better understand their health needs, prioritize risks, and identify locally relevant solutions. This PPI approach, embedded in a One Health framework, offers an alternative to traditional top-down methods, which often overlook the nuanced, community-specific factors influencing health risks in slums.

Four cities—Jaipur, Jakarta, Antofagasta, and Istanbul—were chosen for this study based on their diverse geographical, social, and environmental contexts and their relevance to the CHIP Consortium's focus areas. Each city presents unique challenges related to slum health and One Health, providing a comprehensive overview of how these dynamics play out globally. To implement these interventions effectively, understanding the needs of slum residents through household interviews or surveys is crucial. These methods reveal the fine-grain heterogeneity within and between slums. National and international studies have successfully used this approach. The NIHR Global Health Research Unit on Improving Health in Slums, which maps health services and their uptake across seven slums in Asia and Africa, advocates for household surveys and questionnaires [13]. Our methods across several geographic areas aim to achieve a comprehensive understanding of local health needs and service utilization and this PPI work is intended to inform these interventions.

## 2 Methodology

This CHIP Consortium study employs a PPI approach alongside Community Champions (CC) and a local Community Engagement Team (CET) based in the community who were trained by researchers before the launch of the PPI protocol within the community [15,16]. Team members of the transect walks included local facilitators, observers / Note-takers (if the conversation was not in English, they wrote down anything significant about the household, e.g. number of members and their genders), and Community Champions (CCs). The activities comprise transect walks and social mapping exercises [14] to identify appropriate households for interview and, subsequently, households (mothers with at least one child of less than five years of age) and key informant interviews.

### 2.1 Study sites and participants

The study was conducted in four urban slums located in Jaipur (India), Jakarta (Indonesia), Antofagasta (Chile), and Istanbul (Turkey). These cities were selected based on their varied environmental, social, and economic conditions, providing diverse contexts for exploring One Health challenges [5,6]. Participants included slum residents, local community leaders, healthcare providers, and key informants involved in public health and social services.

Table 1 provides an overview of the diverse contexts of the urban slums selected for the study and details the stakeholders involved in each location to provide a comprehensive perspective on One Health challenges.

### 2.2 Study design

A PPI approach was central to this study, involving community members in all phases of the research, from data collection to interpretation of findings. The primary methods included transect walks, social mapping, and semi-structured interviews [7,15]. These techniques allowed researchers to gather insights directly from the communities, facilitating a bottom-up approach to understanding health risks and designing potential interventions [8,15].

### 2.3 Transect walks

Transect walks were conducted to assess and documents health risks associated with the physical environment of the slums, including water sources, waste management, and areas of environmental contamination. These walks provided an opportunity for community members to guide researchers through their neighborhoods, highlighting specific health risks and challenges including inadequate sanitation, open defecation, accumulation of solid waste and the proximity of animals to human spaces [11]. For example, in Jaipur, transect walks revealed significant environmental contamination in specific areas, particularly near stagnant water bodies where residents dumped waste. The team noted a high presence of stray animals, particularly dogs, contributing to zoonotic risks [8] and AMR due to exposure to improperly disposed antibiotics [17]. Additionally, in Jakarta, the transect walks revealed dense population clusters with no formal waste disposal system, leading to widespread pollution and exposure to untreated wastewater [9,21]. Residents also relied heavily on informal healthcare providers, exacerbating improper antibiotic use [17].

### 2.4 Social mapping

Social mapping exercises were conducted to complement the transect walks by visually representing the distribution of resources and risks within the community, such as healthcare facilities, schools, religious spaces, waste disposal and communal areas. This process also highlighted the presence of community leaders and organisations that could be mobilized for future health interventions [10]. Social mapping in Antofagasta highlighted disparities in access to healthcare services. Certain sections of the slum had no direct access to clinics, leaving residents dependent on local pharmacies for antibiotics without prescriptions. Proximity to industrial areas also exposed residents to heavy pollution, which was strongly linked to respiratory infections and increased use of antibiotics [16,17]. Additionally, In Istanbul, social mapping demonstrated that informal settlements had high animal-human interaction, with residents living in close proximity to livestock. This increased the risk of zoonotic diseases, especially since these animals were not routinely vaccinated, creating a perfect environment for AMR to spread [17].

Table 2 outlines the guidelines used for social mapping, focusing on variables such as topography, population characteristics, and local resources.

### 2.5 Interviews

Semi-structured interviews were conducted with community members and key informants to explore their perceptions of health risks, healthcare access, and the role of local leaders in promoting health [7]. Common concerns raised by residents was about healthcare access, sanitation, and environmental contamination. In Jaipur, residents reported frequent diarrheal outbreaks, often treated with antibiotics from local pharmacies, with no medical supervision. Jakarta interviewees highlighted the precarious conditions of makeshift homes, which were prone to flooding and sewage contamination. Antofagasta residents expressed frustration over poor air quality from nearby industries, which exacerbated respiratory diseases, while in Istanbul, the primary concern was zoonotic diseases related to unvaccinated livestock. Thematic analysis was used to identify key themes related to One Health challenges, as well as potential solutions identified by the community [10,12]. The analysis was guided by the One Health framework, focusing on the social, environmental, and animal health determinants that contribute to AMR and other health challenges [10,13].

### 2.6 Ethical considerations

This study involved pre-ethics PPI activities, such as transect walks, social mapping, and interviews, which were considered exploratory and did not require formal ethical approval. However, ethics approval from the Indian institution was obtained in anticipation of a larger-scale research program proposed for funding, which included more extensive research elements like biological sampling. This approval was secured as a preparatory step to demonstrate readiness and compliance for future research phases, following a common practice in large grant applications. Additionally, informed consent was obtained from all participants, and confidentiality was maintained throughout the research process.

## 3 Results

The findings from this PPI approach through the transect walks and social mapping exercises revealed a significant interconnected health and environmental risks across the four cities, highlighting the importance of a One Health approach. The results are organized into three main categories: environmental, human, and animal health factors, with community insights driving the identification of key themes.

### 3.1 Environmental health factors

Environmental factors such as poor water quality, inadequate sanitation, air pollution, and waste management were critical health determinants across all study sites [11,23]. In Jakarta, contaminated water sources due to industrial waste, which is often shared by animals and humans, were frequently mentioned as contributing to waterborne diseases like diarrheal, additionally, improper waste disposal was also an issue identified [11], while in Jaipur, air pollution from vehicles and industrial activities was a significant concern. Antofagasta's industrial pollution and Istanbul's water access issues also emerged as major environmental health risks [11].

### 3.2 Human health factors

Social determinants, such as overcrowded living conditions, limited access to healthcare, and low health literacy, were common across the slums [12]. In Jaipur and Jakarta, distrust of healthcare professionals due to past experiences of neglect or discrimination was a major barrier to healthcare access, in Antofagasta, residents frequently cited a lack of accessible healthcare services. In some cases, healthcare facilities were distant or difficult to reach, while in others, the cost of services was prohibitive. This was especially problematic for young children, who are particularly vulnerable to infections. Additionally, the inappropriate use of antibiotics was a common issue across all study sites, with many residents reporting that they self-medicated or obtained antibiotics without prescriptions. This practice was identified as a key driver of AMR [17].

### 3.3 Animal health factors

The presence of livestock and vermin in slum areas posed additional health risks, increasing the potential for zoonotic disease transmission. In Jaipur, livestock living in close proximity to humans heightened the risk of zoonotic diseases [24], In Jakarta, residents reported frequent rat infestations, exacerbating sanitation-related health problems [11]. Participants from both Jaipur and Jakarta also noted that animals often defecated in areas where children played, heightening the risk of exposure to pathogens [11].

### 3.4 Community engagement

Community leaders identified through social mapping were seen as key allies in promoting health education and addressing these barriers [19] in both Jaipur and Antofagasta and Istanbul. Additionally, the PPI approach facilitated the identification of local health concerns and priorities for future research. Table 3 presents the themes and subthemes that emerged from the community and key informant interviews. These include environmental contamination, barriers to healthcare access, and the community's desire for follow-up research and involvement in health interventions.

## 4 Discussion

The community-led approach using PPI allowed residents to prioritize the health risks that mattered most to them. Across all four cities, poor sanitation and the spread of infectious diseases were the top concerns. In Jaipur and Jakarta, the risk of zoonotic diseases due to the presence of animals in human living spaces was a particularly urgent issue. In Antofagasta, air pollution and its impact on respiratory health were prioritized, while in Istanbul, water contamination and limited access to clean water were seen as the most pressing problems [19].

The PPI approach in this study offered several advantages over traditional research methods. By involving community members directly in the research process, we were able to capture more nuanced and locally relevant data than might have been possible through external surveys or observational studies alone. This approach fostered trust between researchers and the community, which is critical in slum settings where mistrust of external authorities is common [20].

Additionally, this study demonstrates the value of PPI in understanding One Health challenges in urban slums [18]. By engaging slum residents in the research process, we gained a better understanding of the complex interplay between environmental, human, and animal health risks [22]. Transect walks provided visual data on spatial health risks, while social mapping identified potential community partners for future health interventions [23]. The findings align with other studies emphasizing the importance of community involvement in health research and the need for holistic approaches to address health disparities in marginalized populations [24].

For example, Ravaghi et al. (2021) emphasize that community health needs and assets assessments are crucial for making informed choices about community health. Their findings highlight the need for holistic approaches that consider physical, mental, and social wellbeing, along with broader systemic factors and structural challenges, which is essential for designing effective interventions in slum settings [25]. Similarly, Muhoza et al. (2021) demonstrate the value of key informant perspectives in improving the use of routine health information systems (RHIS) data for decision-making. Their study identifies various sociopolitical, financial, and system design factors that influence RHIS data quality and use [26], which are critical for enhancing health outcomes and tackling AMR. This aligns with the CHIP Consortium's approach of engaging community members to ensure that health interventions are contextually relevant and supported by local stakeholders.

### 4.1 Comparison to traditional approaches

Traditional approaches to addressing health risks in slums often rely on top-down interventions designed by external experts. These interventions frequently fail to account for the social and cultural context of the communities they are intended to help, leading to low uptake and sustainability [21]. In contrast, the PPI approach used in this study empowered community members to identify their own health risks and develop locally relevant solutions, increasing the likelihood that interventions will be accepted and sustained over time [20,22]. The integration of social mapping and transect walks allowed for a more holistic understanding of health risks in slums, as these methods provided real-time data on the interactions between human, environmental, and animal health. This One Health perspective is crucial in slum settings, where health risks are multifactorial and interconnected [23]. For example, the close proximity of animals to human living spaces, combined with poor sanitation, creates a breeding ground for both zoonotic diseases and AMR, which might not have been fully understood without community input [10].

Additionally, the PPI approach demonstrated clear advantages over traditional top-down approaches in these settings:

I. **Cultural Relevance:** Traditional methods often fail to account for local social dynamics and cultural practices [7]. For instance, in Istanbul, previous health interventions did not consider the significance of livestock in residents' livelihoods, leading to low compliance with health guidelines. The PPI approach, by engaging residents in the research process, revealed the need for community-specific solutions [18], such as animal vaccination campaigns that respect local customs.
II. **Trust and Engagement:** In Jaipur, traditional government-led initiatives to improve sanitation had been met with resistance, largely due to a lack of community involvement. By involving residents in transect walks and discussions, the PPI approach fostered trust and allowed for more nuanced, locally acceptable solutions to emerge [20], such as community-led waste management initiatives.
III. **Actionable Insights:** In Jakarta, traditional approaches often overlooked informal healthcare providers, who played a crucial role in antibiotic distribution. Through the PPI approach, we identified the importance of engaging these informal providers in AMR prevention efforts [17], leading to more targeted interventions.
IV. **Tailored Solutions:** The PPI method allowed us to develop tailored interventions, such as the introduction of air quality monitoring programs in Antofagasta and animal health education programs in Istanbul. These solutions emerged from the direct input of community members, ensuring their relevance and sustainability [25].

### 4.2 Importance of the CHIP Consortium

The CHIP Consortium seeks to reframe slums as key areas for Early Warning Systems and economic engines with proper infrastructure and medical support. This is crucial in the context of climate change, population growth, and displacement, which increase reliance on slums and zoonotic disease risk. The research aims to provide cost-effective recommendations for slum upgrades, dependent on centralized support. Residents' hesitancy toward health-related expenditures, potentially leading to late diagnoses and increased antimicrobial resistance (AMR). This study also underscores the need for an integrated One Health approach to address AMR and environmental health risks in marginalized urban settings. The findings suggest that effective One Health interventions must consider the broader social determinants of health, including access to clean water, sanitation, healthcare services, and education [26]. Furthermore, engaging community leaders and residents in the research process proved invaluable in building trust and ensuring that the interventions are culturally sensitive and contextually appropriate [19].

## 5 Conclusion

This PPI-driven study highlights the interconnectedness of environmental, human, and animal health risks in urban slums, emphasizing the need for a One Health approach. The involvement of community members in the research process proved invaluable in identifying local health priorities and potential solutions. Future research should continue to build on these findings by incorporating community-driven interventions and addressing barriers to healthcare access and trust in medical professionals [26].

## Role of the funding source

The workshops & PPI work that led to the development of the conceptual map were funded by the UCL Grand Challenges 2018-19 program, UCL Global Engagement Strategy 2018, UCL-HKU Strategic Partnership Fund, UCL-Knowledge Exchange & Innovation Fund 2018, Aceso Global Health Consultants Pte Ltd., Indian Based Medical Research Council- Global Challenges Research Fund (GCRF) Funding Number: MR/P024114/1/10 and the UCL Santander Research Catalyst Award 2018.

## Participant consent for publication

Informed consent forms for the publication of this PPI study were signed by each participant and stakeholder within this study.

## Ethics approval

Ethical approval for future research activities was granted by the Indian Institute of Public Health. As this manuscript describes pre-ethics PPI activities, no additional ethical approvals were necessary for the other sites involved.

## Data sharing statement

The raw data used to support the findings of this study can be made available upon reasonable request, provided it does not violate the UCL Data Protection Policy.

## CRediT authorship contribution statement

**Logan Manikam:** Supervision, Project administration, Methodology, Funding acquisition, Conceptualization. **Darlington David Faijue:** Writing – review & editing, Writing – original draft, Visualization, Validation, Project administration, Formal analysis, Data curation. **Kalpita Shringarpure:** Writing – review & editing, Writing – original draft, Methodology, Formal analysis, Data curation. **Margi Sheth:** Writing – original draft, Methodology, Investigation. **Pam Factor-Livak:** Writing – review & editing, Validation, Methodology, Investigation, Conceptualization. **Priti Parikh:** Conceptualization, Methodology, Writing – review & editing. **Hector Altamirano-Medina:** Writing – review & editing, Writing – original draft, Methodology, Conceptualization. **Dewi Nur Aisyah:** Writing – original draft, Methodology, Data curation, Conceptualization. **Radhika Sharma:** Writing – review & editing, Writing – original draft, Conceptualization. **Hemant Chaturvedi:** Writing – review & editing, Writing – original draft, Conceptualization. **Kaushik Sarkar:** Writing – review & editing, Writing – original draft, Conceptualization. **Rajib Dasgupta:** Writing – review & editing. **Nancy Hiu Lan Leung:** Writing – review & editing. **Pradeep Kumar Srivastava:** Writing – review & editing. **Monica Lakhanpaul:** Writing – review & editing, Writing – original draft, Visualization, Formal analysis, Conceptualization.

## Declaration of competing interest

The authors declare the following financial interests/personal relationships which may be considered as potential competing interests: Logan Manikam is Director of Aceso Global Health Consultants Pte Limited. Monica Lakhanpaul was supported by the National Institute for Health Research (NIHR) Collaboration for Leadership in Applied Health Research and Care (CLAHRC) North Thames at Bart’s Health NHS Trust. The views expressed are those of the author(s), and are not necessarily those of the NHS, the NIHR, or the Department of Health

## Figures and Tables

**Table 1:** Overview of case study locations and Stakeholders involve in the study.

City	Environmental, Social and Economic conditions	Stakeholders
Jaipur, India	Known for its high population density and limited access to basic sanitation facilities, Jaipur's slums face significant health challenges related to waterborne diseases and waste management. The slums selected for this study primarily consisted of low-income families working in informal sectors such as street vending and construction.	Slum residents, local community leaders, healthcare providers, key public health informants
Jakarta, Indonesia	The urban slums in Jakarta often face flooding, particularly during the monsoon season, which exacerbates health issues such as dengue fever and respiratory infections. The study area included densely populated neighborhoods characterized by makeshift housing, poor waste disposal systems, and limited access to healthcare services.	Slum residents, community leaders, healthcare workers, public health officials
Antofagasta, Chile	Is a desert region, the slums in Antofagasta are shaped by environmental factors such as water scarcity and exposure to industrial pollutants. The residents, many of whom are migrants working in mining and agriculture, face unique health risks related to these harsh environmental conditions.	Migrant workers, community leaders, local health practitioners, environmental and public health informants
Istanbul, Turkey	Diverse populations, composed of refugees, migrants, and low-income residents. These communities experience challenges related to overcrowding, limited access to clean water, and inadequate healthcare infrastructure, making them vulnerable to infectious diseases and other health issues.	Refugees, migrants, local community leaders, healthcare providers, social services and government representatives

**Table 2:** Guidelines for social mapping.

Serial Number	Variables	Observation Features
*Location*		
1	Topography	Mountainous areas, riverine areas, desert areas, natural drainage paths, fall of the land
2	Socio-demographic	High-income areas, low-income areas
3	Housing stock	Sand, cement, mud
*Social Set-Up*		
4	Community leaders	Identify the most influential, knowledgeable, and educated community leaders (religious, elders, elected representatives, etc.)
5	Communal groups	Charity-based organisations, non-governmental organisations, social enterprises, caretakers
6	Population characteristics	Nomadic patterns, any minority groups, refugees
*Local resources*		
7	Communal spaces	Market areas, squares, etc. identify which groups gather there, how often
8	Religious spaces	Mosques, temples, churches
9	Schools	Identify which groups, age-ranges
10	Health facilities (including pharmacies/non-traditional healers)	Which social groups use these facilities
11	Water tanks/pumps, water bodies	Which social groups use these facilities

**Table 3:** Themes and Subthemes emerged from community and key informant Interviews.

Outcome	Theme	Sub-Theme
Validating community need and desire for follow-up research	Need	Lack of pre-existing medical surveillance Barriers to high quality healthcare
	Desire	Higher purpose Appreciation for research Inculcating it in health education practices Technology and hygiene practices; surveillance
Identifying local One Health sources of disease and antimicrobial resistance	Environmental Factors	Water supply and contamination Sanitation Air pollution Housing Solid waste management Energy services Flood water management Roads, natural topography
	Animal Factors	Living alongside livestock Vermin Mosquitos Increase insanitary conditions
	Human Factors	Disease-promoting behaviours and beliefs Intergenerational households Lack of government support Community involvement Leadership skills
Refining CHIP Methodology	Identifying challenges to research	Managing expectations of research Discomfort around biological sampling Scepticism and fearfulness of research efforts and medical professionals Poor medical literacy of slum residents Seasonality of disease
	Adding new lines of enquiry to research	Influence of the father figure Household nutritional intake Allergies Birth defects and intergenerational disease Technology in healthcare Community driven solutions Hesitancy due to expenses involved

## Data Availability

Data will be made available on request.
